# Sufficient Magnesium Intake Reduces Retinal Vein Occlusion Risk: National Health and Nutrition Examination Survey Analysis

**DOI:** 10.3390/nu17071285

**Published:** 2025-04-07

**Authors:** Jiwoo Kim, Min Kim, Christopher Seungkyu Lee, Eun Young Choi

**Affiliations:** 1 Yonsei University College of Medicine, Seoul 03722, Republic of Korea; 2 Department of Ophthalmology, Institute of Vision Research, Gangnam Severance Hospital, Yonsei University College of Medicine, Seoul 06273, Republic of Korea; 3 Department of Ophthalmology, Institute of Vision Research, Severance Eye Hospital, Yonsei University College of Medicine, Seoul 03722, Republic of Korea

**Keywords:** magnesium, retinal vein occlusion, age factor, hypertension, national health and nutrition survey

## Abstract

**Background/Objectives:** Retinal vein occlusion (RVO) is a major cause of vision loss globally. Although magnesium (Mg) is crucial for vascular health, its association with RVO risk is unknown. Thus, we aimed to further examine this association. **Methods:** This cross-sectional study included participants of the Korean National Health and Nutrition Examination Survey 2017–2021 aged ≥19 years (n = 16,358). RVO diagnosis was based on fundus imaging or was self-reported. Based on their daily Mg intake, we categorized participants into low (<120 mg), intermediate (men: 120–300 mg; women: 120–400 mg), and sufficient (men: ≥300 mg; women: ≥400 mg) intake groups and compared their characteristics across groups. **Results:** RVO prevalence was 0.7%. Compared to the non-RVO group, the RVO group was characterized by older individuals, fewer current alcohol consumers, a higher prevalence of hypertension and chronic kidney disease, and a lower intake of fiber, iron, calcium, vitamin E, and Mg. After full adjustment, sufficient Mg intake was significantly associated with a 64% reduced risk of RVO (odds ratio [OR] 0.36, 95% confidence interval [CI] 0.18–0.71, *p* = 0.003). This association was particularly notable among individuals aged 19–59 years (OR 0.18, 95% CI 0.04–0.82, *p* = 0.027), those with hypertension (OR 0.29, 95% CI 0.13–0.67, *p* = 0.003), and those without glaucoma (OR 0.33, 95% CI 0.15–0.71, *p* = 0.004). **Conclusions:** Sufficient Mg intake may reduce RVO risk among adults aged <60 years, individuals with hypertension, and those without glaucoma. Further research should validate the benefits of Mg supplementation in preventing RVO.

## 1. Introduction

Retinal vascular occlusion is one of the principal causes of visual impairment globally. It more frequently affects retinal veins than arteries and generally has a favorable prognosis [1]. In cases of retinal vein occlusion (RVO), more than half of the affected individuals regain functional visual acuity adequate for daily activities with appropriate treatment [2,3]. RVO predominantly affects older adults and has risk factors in common with cardiovascular disease; however, it typically does not necessitate a comprehensive systemic examination [4]. Ocular risk factors for RVO include conditions that impair venous flow, such as ocular hypertension, glaucoma, and retinal arteriolar changes [5,6]. Despite an improved understanding of the pathophysiology of RVO, identifying its modifiable risk factors is imperative to discover potential pathways for preventive and therapeutic interventions.

Magnesium (Mg), an essential dietary mineral, is fundamental to several physiological processes, including blood pressure regulation [7], blood glucose control [8], and nerve function [9]. Its established role in vascular tone and blood pressure regulation and its vasodilatory properties collectively suggest its potential to reduce the risk of cardiovascular disease [10,11]. Nonetheless, the specific relationship between dietary Mg intake and RVO prevalence remains underexplored.

The Korea National Health and Nutrition Examination Survey (KNHANES) provides a comprehensive dataset that includes dietary intake, health outcomes, and a myriad of demographic and lifestyle variables [12]. In this study, leveraging KNHANES data, we aimed to investigate the association between Mg intake and RVO prevalence and to examine the interactions between the effects of Mg intake on RVO risk and other clinical factors. We hypothesized that a higher dietary Mg intake is associated with a lower RVO prevalence.

## 2. Materials and Methods

### 2.1. Data Source and Study Population

The KNHANES is a government-led, population-based, cross-sectional survey designed to evaluate the health and nutritional status of the Korean population and support health policy development [12]. The KNHANES database comprises a wide range of information obtained through questionnaires, such as sociodemographic details, health behaviors, dietary patterns, and medical history, including prevalent ophthalmic conditions. Additionally, biochemical profiles and results from ophthalmological examinations, such as fundus photography, are recorded.

In the present study, we used KNHANES data obtained between 2017 and 2021. Only data of participants aged ≥19 years were included for analysis. Participants with missing ophthalmologic data on RVO or missing nutritional information were excluded.

KNHANES data are publicly available and fully anonymized. Written informed consent was obtained from all participants before their inclusion in the survey. The current study adhered to the tenets of the Declaration of Helsinki. The study protocol was approved by the Institutional Review Board (No. 3-2024-0355).

### 2.2. Nutritional Survey

The nutritional survey conducted as part of the KNHANES 2017–2021 was implemented through in-person interviews by trained medical professionals [13]. The survey encompassed dietary behaviors, feeding frequency, and food intake. Participants were required to provide comprehensive details on all foods and beverages consumed over a 24 h period, including descriptions, quantities, and the timing and location of consumption for each main meal and any additional eating occasions. The daily intake of energy and nutrients was subsequently calculated by applying the reported quantities of all consumed foods and dishes to their respective nutrient values, as obtained from the nutrient database.

### 2.3. Fundus Evaluation

In the 2017–2021 KNHANES, fundus photography was performed under physiological mydriasis conditions to capture color fundus images within a 45° field using VISUCAM 224 (Carl Zeiss Meditec, Jena, Germany). Additionally, posterior segment images were obtained using the Cirrus high-definition optical coherence tomography 500 (Carl Zeiss Meditec). The fundus photographs and optical coherence tomography images were double-graded to diagnose various retinal diseases, including age-related macular degeneration, glaucoma, diabetic retinopathy, RVO, epiretinal membranes, and macular holes [14]. The preliminary grading was performed by ophthalmologists or ophthalmologic residents trained by the Korean Ophthalmologic Society. The final, detailed grading was performed by retinal specialists.

Based on the grading of the fundus and optical coherence tomography images, participants were categorized into non-RVO (without RVO) and RVO groups (with branch or central vein occlusion). RVO was defined by the presence of retinal hemorrhages, dilated veins, cotton wool spots, macular edema, or signs of chronic changes such as collaterals and attenuated veins [6]. Participants who reported having been diagnosed with RVO were also included in the RVO group.

### 2.4. Definition of Variables

In the KNHANES 2017–2021, the dietary pattern survey was based on recall data from the past year, while the nutritional survey focused on the previous 24 h, both relative to the survey time. We incorporated several nutrients, including fiber, dietary micronutrients (iron [Fe], zinc [Zn], calcium [Ca], Mg, β-carotene, vitamin C, vitamin D, and vitamin E), and bioactive compounds, such as ω-3 fatty acids, and identified their beneficial effects in preventing cardiovascular disease [15].

Participants were categorized into three groups according to the daily Mg intake: low (Mg-Low; <120 mg/day; reference group), intermediate (Mg-Int; 120−400 mg for males and 120−300 mg for females), and sufficient intake (Mg-Suff; ≥400 mg for males and ≥300 mg for females). The criteria for group differentiation were based on the recommended dietary allowances outlined by the National Institutes of Health [16].

We defined current alcohol consumption as the consumption of more than one alcoholic drink per month within the preceding year. Participants who had consumed more than 100 cigarettes in their lifetime were classified as smokers. Hypertension was defined by a blood pressure value ≥ 140/90 mmHg or the use of antihypertensive drugs. Diabetes mellitus (DM) was defined by a fasting glucose level ≥126 mg/dL or HbA1c level ≥ 6.5% or the use of hypoglycemic agents and insulin. Participants with a total cholesterol level ≥ 240 mg/dL were diagnosed with dyslipidemia. The criteria for chronic kidney disease (CKD) were an estimated glomerular filtration rate <60 mL/min/1.73 m^2^ or the presence of microalbuminuria; the estimated glomerular filtration rate was calculated using the CKD Epidemiology Collaboration creatinine equation (2021), and the criterion for microalbuminuria was a urine albumin-to-creatinine ratio ≥30 μg/mg. Finally, patients with hematocrit ≥54% for males and ≥48% for females were diagnosed with polycythemia.

### 2.5. Statistical Analysis

To compare baseline characteristics, we used Student’s t-test for continuous variables and the chi-squared test for categorical variables. To assess the association between Mg intake and RVO risk, we employed multiple logistic regression analyses and estimated the odds ratio (OR) for RVO with adjustment for multiple covariates. Adjusted covariates included the following: sex; age; alcohol intake and smoking status; body mass index; comorbidities (hypertension, DM, dyslipidemia, CKD, and polycythemia); the presence of glaucoma; and nutrient intake, including dietary fiber, Fe, Zn, Ca, β-carotene, vitamin C, vitamin D, vitamin E, ω-3 fatty acids, and Mg. We also conducted subgroup analyses based on interaction tests to determine whether the effect of Mg intake remained consistent across various clinical situations.

All statistical analyses were conducted using IBM SPSS Statistics version 22.0 (IBM Corp., Armonk, NY, USA) and R software version 3.6.3 (R Project for Statistical Computing, Vienna, Austria). *p*-values less than 0.05 indicated statistical significance.

To evaluate whether the sample size was sufficient for statistical analysis, we conducted an a priori power analysis using G*Power version 3.1. Since no prior studies reported a reliable OR for the association between magnesium intake and RVO, we assumed that higher magnesium intake would reduce the risk of RVO by half (OR = 0.5). Based on an expected RVO prevalence of 0.7% (Pr(Y = 1|X = 1) = 0.007), significance level of 0.05, power of 0.80, and R^2^ of other predictors = 0.1, the required total sample size was estimated to be approximately 10,500.

## 3. Results

**Figure 1:**
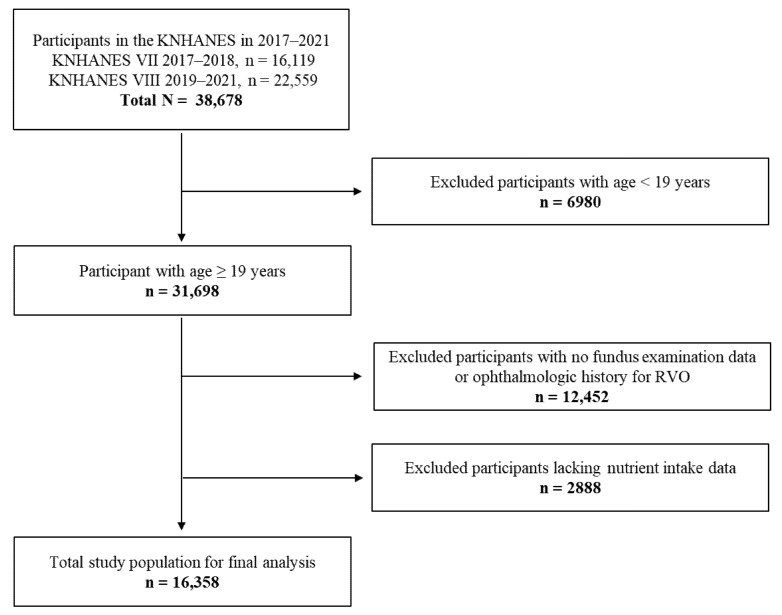
Flowchart of participant selection.

Of the total 38,678 KNHANES participants (cycle 2017‒2018: n = 16,119; cycle 2019‒2021: n = 22,559), after the exclusion of those aged <19 years (n = 6980) and those with missing ophthalmologic data (n = 12,452) and missing nutrient intake data (n = 2888), 16,358 participants were included in the analysis (Figure 1). Among them, 118 participants were allocated to the RVO group: 99 diagnosed with RVO based on fundus photography and 19 who self-reported having RVO.

### 3.1. Baseline Characteristics

Participants’ baseline characteristics are shown in Table 1. Compared to the non-RVO group, the RVO group comprised older individuals; a lower proportion of current alcohol consumers; a higher proportion of individuals with hypertension, CKD, and glaucoma; and individuals with a lower intake of dietary fiber, Fe, Ca, vitamin E, and Mg.

The proportions of male participants and smokers were higher in the RVO group than in the non-RVO group; however, the difference was not significant. The RVO group also had a higher prevalence of DM, lower prevalence of hyperlipidemia and polycythemia, and lower intakes of Zn, β-carotene, vitamin C, vitamin D, and ω-3 fatty acids; these differences were not significant.

### 3.2. Association Between Mg Intake and RVO Risk

**Figure 2:**
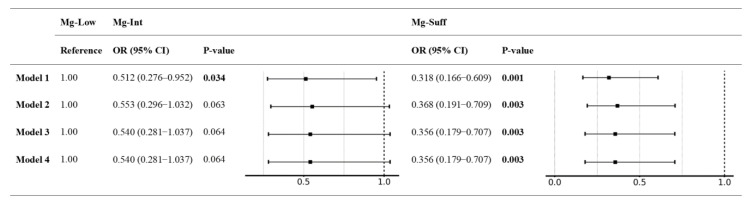
Risk of retinal vein occlusion stratified by daily magnesium intake. Model 1: unadjusted. Model 2: adjusted for age, sex, and BMI. Model 3: adjusted for model 2 covariates plus smoking, alcohol consumption, and comorbidities. Model 4: adjusted for model 3 covariates plus daily nutrient intake. Bold font for *p*-value indicates statistical significance. OR—odds ratio; CI—confidence interval; BMI—body mass index.

**Figure 3:**
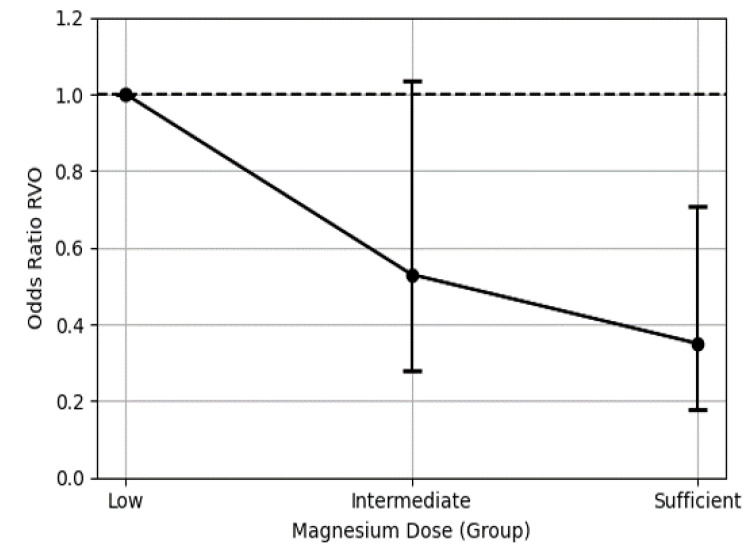
Dose–response analyses of the association between dietary magnesium intake and the risk of retinal vein occlusion. Error bars indicate confidence intervals.

In the unadjusted model, higher levels of Mg intake were progressively associated with a reduced RVO risk compared to that of the Mg-Low group (Mg-Int: OR 0.51, 95% confidence interval [CI] 0.28–0.95, *p* = 0.034; Mg-Suff: OR 0.32, 95% CI 0.17–0.61, *p* = 0.001; model 1; Figure 2). The relationship between increased Mg intake levels and a progressive reduction in RVO risk remained consistent after adjustment for sex, age, and body mass index (Mg-Int: OR 0.55, 95% CI 0.30–1.03, *p* = 0.063; Mg-Suff: OR 0.37, 95% CI 0.19–0.71, *p* = 0.003; model 2; Figure 2), as well as after further adjustment for alcohol consumption and smoking, hypertension, DM, dyslipidemia, CKD, and polycythemia (Mg-Int: OR 0.54, 95% CI 0.28–1.04, *p* = 0.064; Mg-Suff: OR 0.36, 95% CI 0.18–0.71, *p* = 0.003; model 3; Figure 2). Even after further adjustment for nutrient intake, the association between Mg intake and reduced RVO risk persisted (Mg-Int: OR 0.54, 95% CI 0.28–1.04, *p* = 0.064; Mg-Suff: OR 0.36, 95% CI 0.18–0.71, *p* = 0.003; model 4; Figure 2). In the final adjusted model, age (OR 1.03, 95% CI 1.01–1.05, *p* < 0.002), hypertension (OR 2.49, 95% CI 1.60–3.88, *p* < 0.001), and glaucoma (OR 3.49, 95% CI 2.12–5.76, *p* < 0.001) were associated with an increased risk of RVO. Table S1 presents the ORs for all covariates included in each model. The final adjusted model demonstrated an inverse correlation between Mg intake and RVO risk, as illustrated in the dose–response graph (Figure 3).

### 3.3. Stratified Analyses

We performed subgroup analyses according to age and the presence of hypertension and glaucoma, which were associated with RVO development. *p*-values for interaction were all greater than 0.05 (Table S2). Given that age, hypertension, and glaucoma are significant risk factors for RVO development and progression [5,17], we conducted a further sensitivity analysis for each stratified condition.

**Figure 4:**
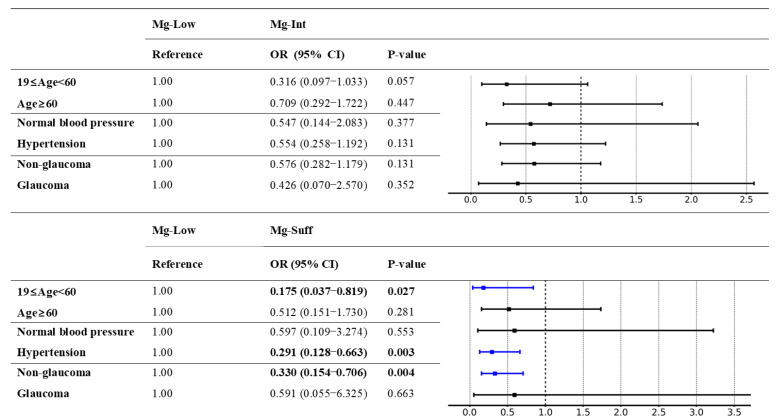
Forest plots of odds ratios for retinal vein occlusion associated with magnesium intake, stratified by age and presence of hypertension and glaucoma. Bold font for *p*-value and blue error bars in the forest plot indicate statistical significance. OR—odds ratio; CI—confidence interval.

We divided the entire sample into two age groups: 19‒60 and ≥ 60 years (Figure 4 and Table S3). In the 19‒60-year age group, both intermediate (OR 0.32, 95% CI 0.10–1.03, *p* = 0.057) Mg intake and sufficient (OR 0.18, 95% CI 0.04–0.82, *p* = 0.027) Mg intake were progressively associated with reduced RVO risk. In the ≥60-year age group, daily Mg intake was not significantly associated with RVO risk (Mg-Int: OR 0.71, 95% CI 0.29–1.72, *p* = 0.447; Mg-Suff: OR 0.51, 95% CI 0.15–1.73, *p* = 0.281).

Next, the sample was categorized into two groups based on the presence of hypertension (Figure 4 and Table S4). In the normal blood pressure group, RVO was not significantly associated with daily Mg intake (Mg-Int: OR 0.55, 95% CI 0.14–2.08, *p* = 0.377; Mg-Suff: OR 0.60, 95% CI 0.11–3.28, *p* = 0.553). In the group with hypertension, sufficient Mg intake was significantly associated with reduced RVO risk (Mg-Int: OR 0.57, 95% CI 0.27–1.22, *p* = 0.149; Mg-Suff: OR 0.29, 95% CI 0.13–0.67, *p* = 0.003).

Finally, we stratified the sample based on the presence of glaucoma (Figure 4 and Table S5). In the non-glaucoma group, sufficient Mg intake was significantly associated with reduced RVO risk (Mg-Int: OR 0.58, 95% CI 0.28–1.18, *p* = 0.131; Mg-Suff: OR 0.33, 95% CI 0.15–0.71, *p* = 0.004). Conversely, in the glaucoma group, sufficient Mg intake was not significantly associated with RVO risk (Mg-Int: OR 0.43, 95% CI 0.07–2.57, *p* = 0.352; Mg-Suff: OR 0.59, 95% CI 0.06–6.33, *p* = 0.663).

## 4. Discussion

In this nationwide population-based study, we examined the association between Mg intake and RVO prevalence among KNHANES participants. Sufficient Mg intake was associated with a 64% reduction in the odds of RVO, particularly among individuals aged <60 years, those with hypertension, and those without glaucoma. This underscores the potential of Mg intake as a modifiable risk factor for RVO, highlighting the importance of dietary recommendations in the prevention of this condition. Insights from this study may help inform preventive nutritional strategies for RVO.

The association between Mg intake and decreased RVO risk may be attributed to magnesium’s role in maintaining vascular health and blood pressure homeostasis. Mg is known to influence vascular tone [18], blood pressure regulation [7,19], and endothelial function [20,21], all of which are critical factors in the pathogenesis of vascular occlusions. Furthermore, Mg deficiency has been linked to the development of atherosclerosis, which may promote vascular calcification and lipid accumulation in vascular plaques [22,23]. Anti-inflammatory effects of Mg in reducing serum C-reactive protein concentrations have also been reported [24]. Blood pressure regulation, vascular tone, inflammation, and atherosclerosis are closely interconnected, with endothelial dysfunction serving as a central factor. These processes are also linked to coagulation pathways, which may be relevant to the development of RVO. Although magnesium has been shown to affect several aspects of vascular function, the exact biological mechanisms by which it influences RVO development remain unclear and warrant further research.

The protective association between magnesium intake and RVO may be more pronounced among individuals aged 19–59 years and those with hypertension. While the exact mechanisms remain unclear and require further investigation, we speculate that younger individuals may benefit more from the protective effects of magnesium due to greater vascular plasticity, better endothelial function, and less cumulative vascular damage. Similarly, individuals with hypertension may experience stronger effects, as magnesium is known to regulate vascular tone, blood pressure, and systemic inflammation. Multiple meta-analyses of prospective trials have shown that chronic insufficient Mg intake is associated with an increased risk of several clinical conditions, including hypertension [7], cardiovascular disease [25], and stroke [26].

In addition to age, hypertension, and glaucoma, which are well-known risk factors for RVO, our study identified fiber intake as a possible independent risk factor for RVO. To the best of our knowledge, this is the first study to report this association. A systemic review and meta-analysis of prospective trials showed that a high-fiber diet lowers blood pressure and reduces the risk of cardiovascular disease [27]. In this context, a higher fiber intake was significantly associated with reduced RVO risk. However, the OR was not sufficiently low to establish its clinical significance, indicating the need for further research on this subject.

A strength of our study is the use of well-documented data from a national survey, which used detailed questionnaires on dietary behavior and ophthalmological history. This enabled us to reliably establish an association between Mg intake and RVO risk in a large population. However, this study has certain limitations. First, the cross-sectional design of the study limits the establishment of causality between magnesium intake and RVO risk. As the analysis was based on previously diagnosed RVO cases and lacked longitudinal follow-up, temporal associations could not be assessed. Therefore, the observed inverse association between sufficient magnesium intake and RVO risk should be interpreted with caution. Further longitudinal cohort studies or randomized controlled trials are needed to confirm these findings, determine the optimal dosage and safety profile of magnesium intake, and explore its potential preventive effect in high-risk populations and across diverse subgroups. Second, given the relatively small proportion of participants with RVO, residual confounding—such as unexamined inflammatory conditions, genetic predispositions, or other dietary and lifestyle factors—may still be present and could have influenced the observed associations, despite adjustment for key variables. Therefore, the effect of magnesium should be interpreted as part of the broader multifactorial influences on RVO. The third limitation of this study arises from the constraints of the KNHANES dataset. Blood Mg concentrations were not analyzed, and it was not feasible to distinguish between RVO and arterial occlusions. Additionally, ocular risk factors for RVO and distinctions between RVO subtypes (branch vs. central, ischemic vs. non-ischemic) were not accounted for in the analysis. Both dietary Mg intake and RVO diagnosis—in 19 cases where fundus grading was not available—were assessed through self-reported surveys, which may have introduced recall bias and potential misclassification. In addition, because the study population was limited to Korean adults, the generalizability of our findings to other ethnic or regional populations may be limited.

## 5. Conclusions

Sufficient Mg intake was associated with a significantly lower risk of RVO. This highlights the potential of sufficient dietary Mg intake as a preventive measure against RVO in adults, particularly those aged <60 years, individuals with hypertension, and those without glaucoma. Future prospective studies should confirm the protective effect of Mg against RVO and explore its underlying mechanisms.

## Figures and Tables

**Table 1:** General sociodemographic and clinical characteristics of the study population stratified by the presence of retinal vein occlusion.

Characteristic	Non-RVO (N = 16,240)	RVO (N = 118)	*p*-Value
Male (vs. female)	6796 (41.8)	52 (44.1)	0.626
Age, years	57.32 ± 0.107	64.81 ± 1.095	**<0.001**
Body mass index, kg/m^2^	24.08 ± 0.027	24.70 ± 0.314	0.050
Current alcohol consumption, yes (vs. no)	7831 (48.5)	46 (39.0)	**0.040**
Lifetime smoker (vs. nonsmoker)	2478 (15.4)	19 (16.1)	0.822
Comorbidities based on biochemical profiles			
Hypertension, yes (vs. no)	6156 (38.0)	82 (70.1)	**<0.001**
Diabetes mellitus, yes (vs. no)	2835 (17.8)	28 (24.3)	0.069
Dyslipidemia, yes (vs. no)	1805 (11.4)	9 (7.9)	0.242
Chronic kidney disease, yes (vs. no)	589 (3.8)	9 (8.1)	**0.018**
Polycythemia, yes (vs. no)	87 (0.5)	0 (0.0)	0.427
Glaucoma, yes (vs. no)	744 (4.6)	24 (20.3)	**<0.001**
Nutrient intake per day			
Dietary fiber intake, g	26.58 ± 0.112	22.89 ± 1.151	**0.005**
Iron intake, mg	10.46 ± 0.052	8.94 ± 0.455	**0.014**
Zinc intake, mg	9.99 ± 0.041	9.08 ± 0.448	0.062
Calcium intake, mg	495.53 ± 2.348	436.63 ± 25.179	**0.033**
β-carotene intake, μg	2954.87 ± 23.295	2862.05 ± 285.151	0.735
Vitamin C intake, mg	64.61 ± 0.718	54.30 ± 5.626	0.222
Vitamin D intake, μg	2.99 ± 0.046	2.45 ± 0.319	0.327
Vitamin E intake, mg	6.42 ± 0.031	5.45 ± 0.303	**0.008**
ω-3 fatty acid intake, g	1.81 ± 0.016	1.56 ± 0.137	0.175
Magnesium intake, mg	313.94 ± 1.153	280.5 ± 12.822	**0.013**

Values are presented as numbers (percentages) or mean ± standard error. Bold font for *p*-value indicates statistical significance. RVO—retinal vascular occlusion.

## Data Availability

The raw data are available at the KNHANES website (https://knhanes.cdc.go.kr, accessed on 1 March 2025).

## Supplementary Materials

The following supporting information can be downloaded at https://www.mdpi.com/article/10.3390/nu17071285/s1, Table S1: Magnesium intake as an independent predictive factor for a decreased risk of retinal vein occlusion in the multiple logistic regression analysis; Table S2: Interaction *p*-values for each variable with magnesium intake; Table S3: Subgroup analysis of the association between daily magnesium intake and the risk of retinal vein occlusion according to age; Table S4: Subgroup analysis of the association between daily magnesium intake and the risk of retinal vein occlusion according to the presence of hypertension; Table S5: Subgroup analysis of the association between daily magnesium intake and the risk of retinal vein occlusion according to the presence of glaucoma.

## Abbreviations

The following abbreviations are used in this manuscript:

Ca	Calcium
CI	Confidence interval
CKD	Chronic kidney disease
DM	Diabetes mellitus
Fe	Iron
KNHANES	Korea National Health and Nutrition Examination Survey
Mg	Magnesium
Mg-Low	Low magnesium intake
Mg-Int	Intermediate magnesium intake
Mg-Suff	Sufficient magnesium intake
OR	Odds ratio
RVO	Retinal vein occlusion
Zn	Zinc
