# Supplementary material for: Sufficient Magnesium Intake Reduces Retinal Vein Occlusion Risk: National Health and Nutrition Examination Survey Analysis
Source: Nutrients. 2025 Apr 7;17(7):1285. doi: 10.3390/nu17071285 (PMC11990526; doi:10.3390/nu17071285)
Supplement: Supplementary file 1 [file nutrients-17-01285-s001.zip › RVO_Mg_Table_S1_250304.pdf]

Table S1. Magnesium intake as an independent predictive factor for decreased risk of retinal vein occlusion in the multiple logistic regression analysis

Variables	Model 1		Model 2		Model 3		Model 4	
	OR (95% CI)	*p*-value	OR (95% CI)	*p*-value	OR (95% CI)	*p*-value	OR (95% CI)	*p*-value
**Male (vs female)**			1.269 (0.868–1.853)	0.218	1.266 (0.812–1.975)	0.297	1.258 (0.804–1.970)	0.314
**Age, years**			**1.043 (1.028–1.058)**	**<0.001**	**1.027 (1.010–1.045)**	**0.002**	**1.027 (1.010–1.045)**	**0.002**
**Body mass index, kg/m^2**			**1.055 (1.001–1.111)**	**0.046**	1.043 (0.986–1.104)	0.143	1.042 (0.985–1.103)	0.152
**Current alcohol consumption, yes (vs no)**					0.776 (0.502–1.199)	0.253	0.774 (0.500–1.198)	0.251
**Lifetime smoker (vs nonsmoker)**					1.223 (0.692–2.162)	0.489	1.184 (0.668–2.101)	0.563
**Hypertension, yes (vs no)**					**2.494 (1.602–3.882)**	**<0.001**	**2.494 (1.602–3.882)**	**<0.001**
**Diabetes mellitus, yes (vs no)**					0.816 (0.511–1.303)	0.394	0.826 (0.517–1.320)	0.425
**Dyslipidemia, yes (vs no)**					0.803 (0.402–1.602)	0.533	0.794 (0.397–1.584)	0.512
**Chronic kidney disease, yes (vs no)**					0.898 (0.422–1.910)	0.780	0.885 (0.416–1.886)	0.752
**Polycythemia, yes (vs no)**					0.000 (0.000–0.000)	0.997	0.000 (0.000–0.000)	0.997
**Glaucoma, yes (vs no)**					**3.493 (2.118–5.761)**	**<0.001**	**3.493 (2.118–5.761)**	**<0.001**
**Dietary fiber intake, g**							0.982 (0.959–1.006)	0.144
**Iron intake, g**							0.980 (0.929–1.034)	0.459
**Zinc intake, g**							1.023 (0.983–1.066)	0.258
**Calcium intake, mg**							1.000 (0.999–1.001)	0.811
**β-carotene intake, μg**							1.000 (1.000–1.000)	0.236
**Vitamin C intake, mg**							1.000 (0.997–1.003)	0.870
**Vitamin D intake, μg**							0.993 (0.948–1.040)	0.759
**Vitamin E intake, mg**							1.023 (0.934–1.120)	0.625
**ω-3 fatty acids intake, g**							1.007 (0.885–1.145)	0.921
**Mg intake**								
**Mg-Low**	1 (reference)		1 (reference)		1 (reference)		1 (reference)	
**Mg-Int**	**0.512 (0.276–0.952)**	**0.034**	0.553 (0.296–1.032)	0.063	0.540 (0.281–1.037)	0.064	0.540 (0.281–1.037)	0.064
**Mg-Suff**	**0.318 (0.166–0.609)**	**0.001**	**0.368 (0.191–0.709)**	**0.003**	**0.356 (0.179–0.707)**	**0.003**	**0.356 (0.179–0.707)**	**0.003**

Model 1: Unadjusted.

Model 2: Adjusted for age, sex, and BMI.
Model 3: Adjusted for model 2 covariates plus smoking, alcohol consumption, and comorbidities.
Model 4: Adjusted for model 3 covariates plus daily nutrient intake
Bold font in *p*-value indicates statistical significance. OR, odds ratio; CI, confidence interval
