# Supplementary material for: Sufficient Magnesium Intake Reduces Retinal Vein Occlusion Risk: National Health and Nutrition Examination Survey Analysis
Source: Nutrients. 2025 Apr 7;17(7):1285. doi: 10.3390/nu17071285 (PMC11990526; doi:10.3390/nu17071285)
Supplement: Supplementary file 1 [file nutrients-17-01285-s001.zip › RVO_Mg_Table_S2_250304.pdf]

Table S2. Interaction *p*-values for each variable with magnesium intake

**Variables**	***p* for interaction**
**Male (vs female)**	0.320
**Age, years**	0.871
**Body mass index, kg/m^2**	0.974
**Current alcohol consumption, yes (vs no)**	0.919
**Lifetime smoker (vs nonsmoker)**	0.339
**Hypertension, yes (vs no)**	0.391
**Diabetes mellitus, yes (vs no)**	0.383
**Dyslipidemia, yes (vs no)**	0.097
**Chronic kidney disease, yes (vs no)**	0.390
**Polycythemia, yes (vs no)**	1.000
**Glaucoma, yes (vs no)**	0.222
**Dietary fiber intake, g**	0.946
**Iron intake, g**	0.464
**Zinc intake, g**	0.482
**Calcium intake, mg**	0.448
**β-carotene intake, μg**	0.790
**Vitamin C intake, mg**	0.340
**Vitamin D intake, μg**	0.726
**Vitamin E intake, mg**	0.522
**ω-3 fatty acids intake, g**	0.632
