# Supplementary material for: Sufficient Magnesium Intake Reduces Retinal Vein Occlusion Risk: National Health and Nutrition Examination Survey Analysis
Source: Nutrients. 2025 Apr 7;17(7):1285. doi: 10.3390/nu17071285 (PMC11990526; doi:10.3390/nu17071285)
Supplement: Supplementary file 1 [file nutrients-17-01285-s001.zip › RVO_Mg_Table_S3_250304.pdf]

Table S3. Subgroup analysis of the association between daily magnesium intake and the risk of retinal vein occlusion according to age

Variables	19≤Age<60		Age≥60	
	OR (95% CI)	*p*-value	OR (95% CI)	*p*-value
**Male (vs female)**	1.486 (0.670–3.296)	0.330	1.130 (0.644–1.984)	0.670
**Age, years**	1.037 (0.984–1.092)	0.176	1.020 (0.981–1.060)	0.321
**Body mass index, kg/m^2**	1.061 (0.969–1.162)	0.198	1.025 (0.952–1.103)	0.513
**Current alcohol consumption, yes (vs no)**	0.733 (0.359–1.493)	0.392	0.791 (0.452–1.382)	0.409
**Lifetime smoker (vs nonsmoker)**	0.849 (0.338–2.130)	0.704	1.393 (0.669–2.900)	0.376
**Hypertension, yes (vs no)**	**3.353 (1.682–6.686)**	**<0.001**	**2.157 (1.263–3.683)**	**0.005**
**Diabetes mellitus, yes (vs no)**	0.821 (0.312–2.155)	0.688	0.804 (0.469–1.379)	0.429
**Dyslipidemia, yes (vs no)**	1.054 (0.403–2.755)	0.914	0.586 (0.211–1.633)	0.307
**Chronic kidney disease, yes (vs no)**	**10.632 (3.502–32.279)**	**<0.001**	0.462 (0.164–1.303)	0.144
**Polycythemia, yes (vs no)**	0.000 (0.000–0.000)	0.998	0.000 (0.000–0.000)	0.998
**Glaucoma, yes (vs no)**	2.445 (0.709–8.433)	0.157	**3.834 (2.207–6.662)**	**<0.001**
**Dietary fiber intake, g**	0.991 (0.953–1.031)	0.659	**0.972 (0.953–0.992)**	**0.006**
**Iron intake, mg**	0.992 (0.921–1.069)	0.842	0.966 (0.897–1.041)	0.365
**Zinc intake, mg**	1.011 (0.942–1.085)	0.761	1.053 (0.971–1.143)	0.212
**Calcium intake, mg**	1.001 (0.999–1.002)	0.241	1.000 (0.998–1.001)	0.569
**β-carotene intake, µg**	1.000 (1.000–1.000)	0.105	1.000 (1.000–1.000)	0.855
**Vitamin C intake, mg**	1.000 (0.995–1.004)	0.870	1.001 (0.997–1.005)	0.609
**Vitamin D intake, µg**	1.020 (0.961–1.082)	0.518	0.965 (0.894–1.042)	0.366
**Vitamin E intake, mg**	0.975 (0.853–1.113)	0.705	1.065 (0.943–1.203)	0.310
**ω-3 fatty acids intake, g**	0.947 (0.733–1.223)	0.676	1.042 (0.893–1.217)	0.598
**Mg intake**				
**Mg-Low**	1 (reference)		1 (reference)	
**Mg-Int**	0.316 (0.097–1.033)	0.057	0.709 (0.292–1.722)	0.447
**Mg-Suff**	**0.175 (0.037–0.819)**	**0.027**	0.512 (0.151–1.730)	0.281

Bold font in *p*-value indicates statistical significance. OR, odds ratio; CI, confidence interval,.
