# Supplementary material for: Sufficient Magnesium Intake Reduces Retinal Vein Occlusion Risk: National Health and Nutrition Examination Survey Analysis
Source: Nutrients. 2025 Apr 7;17(7):1285. doi: 10.3390/nu17071285 (PMC11990526; doi:10.3390/nu17071285)
Supplement: Supplementary file 1 [file nutrients-17-01285-s001.zip › RVO_Mg_Table_S4_250304.pdf]

Table S4. Subgroup analysis of the association between daily magnesium intake and the risk of retinal vein occlusion according to the presence of hypertension

Variables	Hypertension		Normal blood pressure	
	OR (95% CI)	*p*-value	OR (95% CI)	*p*-value
**Male (vs female)**	0.951 (0.545–1.660)	0.860	1.763 (0.788–3.944)	0.167
**Age, years**	1.018 (0.994–1.042)	0.151	**1.051 (1.022–1.081)**	**<0.001**
**Body mass index, kg/m^2**	1.041 (0.974–1.113)	0.235	1.027 (0.919–1.147)	0.640
**Current alcohol consumption, yes (vs no)**	1.060 (0.619–1.815)	0.831	0.468 (0.216–1.011)	0.053
**Lifetime smoker (vs nonsmoker)**	0.754 (0.338–1.679)	0.489	**2.159 (1.001–4.657)**	**0.050**
**Hypertension, yes (vs no)**				
**Diabetes mellitus, yes (vs no)**	0.877 (0.520–1.479)	0.622	0.692 (0.234–2.047)	0.506
**Dyslipidemia, yes (vs no)**	0.544 (0.196–1.513)	0.243	1.153 (0.440–3.018)	0.772
**Chronic kidney disease, yes (vs no)**	0.982 (0.434–2.221)	0.964	1.000 (0.125–7.978)	1.000
**Polycythemia, yes (vs no)**	0.000 (0.000–0.000)	0.998	0.000 (0.000–0.000)	0.998
**Glaucoma, yes (vs no)**	**4.202 (2.418–7.300)**	**<0.001**	1.899 (0.565–6.377)	0.299
**Dietary fiber intake, g**	0.987 (0.958–1.016)	0.371	0.977 (0.937–1.018)	0.263
**Iron intake, mg**	0.970 (0.902–1.043)	0.406	0.995 (0.917–1.079)	0.895
**Zinc intake, mg**	**1.078 (1.001–1.160)**	**0.047**	0.961 (0.851–1.085)	0.520
**Calcium intake, mg**	0.999 (0.997–1.000)	0.089	**1.001 (1.000–1.002)**	**0.009**
**β-carotene intake, µg**	1.000 (1.000–1.000)	0.142	1.000 (1.000–1.000)	0.994
**Vitamin C intake, mg**	1.001 (0.996–1.005)	0.778	1.000 (0.995–1.005)	0.968
**Vitamin D intake, µg**	1.006 (0.952–1.063)	0.836	0.965 (0.873–1.067)	0.491
**Vitamin E intake, mg**	1.064 (0.951–1.189)	0.278	0.982 (0.842–1.147)	0.822
**ω-3 fatty acids intake, g**	0.938 (0.758–1.161)	0.555	1.050 (0.963–1.145)	0.270
**Mg intake**				
**Mg-Low**	1 (reference)		1 (reference)	
**Mg-Int**	0.554 (0.258–1.192)	0.131	0.547 (0.144–2.083)	0.377
**Mg-Suff**	**0.291 (0.128–0.663)**	**0.003**	0.597 (0.109–3.274)	0.553

Bold font in *p*-value indicates statistical significance. OR, odds ratio; CI, confidence interval.
