# Supplementary material for: Sufficient Magnesium Intake Reduces Retinal Vein Occlusion Risk: National Health and Nutrition Examination Survey Analysis
Source: Nutrients. 2025 Apr 7;17(7):1285. doi: 10.3390/nu17071285 (PMC11990526; doi:10.3390/nu17071285)
Supplement: Supplementary file 1 [file nutrients-17-01285-s001.zip › RVO_Mg_Table_S5_250304.pdf]

Table S5. Subgroup analysis of the association between daily magnesium intake and the risk of retinal vein occlusion according to the presence of glaucoma

Variables	Glaucoma		Non-glaucoma	
	OR (95% CI)	*p*-value	OR (95% CI)	*p*-value
**Male (vs female)**	1.786 (0.574–5.555)	0.317	1.161 (0.706–1.907)	0.556
**Age, years**	1.041 (0.990–1.095)	0.115	**1.027 (1.008–1.046)**	**0.004**
**Body mass index, kg/m^2**	1.100 (0.963–1.278)	0.149	1.031 (0.969–1.098)	0.337
**Current alcohol consumption, yes (vs no)**	0.956 (0.327–2.794)	0.935	0.715 (0.440–1.162)	0.176
**Lifetime smoker (vs nonsmoker)**	0.926 (0.238–3.601)	0.912	1.276 (0.675–2.410)	0.453
**Hypertension, yes (vs no)**	**4.974 (1.444–17.126)**	**0.011**	**2.329 (1.443–3.758)**	**<0.001**
**Diabetes mellitus, yes (vs no)**	0.295 (0.081–1.079)	0.065	1.019 (0.614–1.691)	0.943
**Dyslipidemia, yes (vs no)**	1.020 (0.212–4.908)	0.981	0.749 (0.344–1.634)	0.468
**Chronic kidney disease, yes (vs no)**	0.920 (0.185–4.578)	0.918	0.876 (0.367–2.090)	0.765
**Polycythemia, yes (vs no)**	0.000 (0.000–0.000)	0.999	0.000 (0.000–0.000)	0.997
**Glaucoma, yes (vs no)**				
**Dietary fiber intake, g**	0.995 (0.941–1.053)	0.871	0.976 (0.937–1.017)	0.256
**Iron intake, mg**	0.922 (0.773–1.100)	0.368	0.979 (0.953–1.005)	0.117
**Zinc intake, mg**	1.043 (0.878–1.238)	0.634	1.020 (0.979–1.063)	0.348
**Calcium intake, mg**	0.999 (0.996–1.002)	0.556	1.000 (0.999–1.001)	0.566
**β-carotene intake, µg**	1.000 (1.000–1.000)	0.326	1.000 (1.000–1.000)	0.385
**Vitamin C intake, mg**	1.000 (0.990–1.010)	0.986	1.000 (0.997–1.003)	0.824
**Vitamin D intake, µg**	1.002 (0.878–1.144)	0.974	0.991 (0.942–1.042)	0.727
**Vitamin E intake, mg**	0.994 (0.800–1.235)	0.955	1.026 (0.930–1.133)	0.604
**ω-3 fatty acids intake, g**	1.068 (0.776–1.469)	0.687	1.001 (0.867–1.155)	0.994
**Mg intake**				
**Mg-Low**	1 (reference)		1 (reference)	
**Mg-Int**	0.426 (0.070–2.570)	0.352	0.576 (0.282–1.179)	0.131
**Mg-Suff**	0.591 (0.055–6.325)	0.663	**0.330 (0.154–0.706)**	**0.004**

Bold font in *p*-value indicates statistical significance. OR, odds ratio; CI, confidence interval.
